# A Prototype Method for the Detection and Recognition of Pigments in the Environment Based on Optical Property Simulation

**DOI:** 10.3390/plants12244178

**Published:** 2023-12-15

**Authors:** Roman Y. Pishchalnikov, Denis D. Chesalin, Vasiliy A. Kurkov, Uliana A. Shkirina, Polina K. Laptinskaya, Vasiliy S. Novikov, Sergey M. Kuznetsov, Andrei P. Razjivin, Maksim N. Moskovskiy, Alexey S. Dorokhov, Andrey Yu. Izmailov, Sergey V. Gudkov

**Affiliations:** 1 Prokhorov General Physics Institute of the Russian Academy of Sciences, 119991 Moscow, Russia; genoa-and-pittsburgh@mail.ru (D.D.C.); v.k27@yandex.ru (V.A.K.); shkirinajuliana@gmail.com (U.A.S.); polinalaptinskaya@gmail.com (P.K.L.); vs.novikov@kapella.gpi.ru (V.S.N.); kuznetsovsm@kapella.gpi.ru (S.M.K.); s_makariy@rambler.ru (S.V.G.); 2 Belozersky Research Institute of Physico-Chemical Biology, Moscow State University, 119992 Moscow, Russia; razjivin@belozersky.msu.ru; 3 Federal State Budgetary Scientific Institution “Federal Scientific Agroengineering Center VIM” (FSAC VIM), 109428 Moscow, Russia; maxmoskovsky74@yandex.ru (M.N.M.); dorokhov.vim@yandex.ru (A.S.D.); vim@vim.ru (A.Y.I.)

**Keywords:** optimization, differential evolution, absorption, optical response, carotenoids, multimode Brownian oscillator model, fungal infection, *Fusarium graminearum*

## Abstract

The possibility of pigment detection and recognition in different environments such as solvents or proteins is a challenging, and at the same time demanding, task. It may be needed in very different situations: from the nondestructive in situ identification of pigments in paintings to the early detection of fungal infection in major agro-industrial crops and products. So, we propose a prototype method, the key feature of which is a procedure analyzing the lineshape of a spectrum. The shape of the absorption spectrum corresponding to this transition strongly depends on the immediate environment of a pigment and can serve as a marker to detect the presence of a particular pigment molecule in a sample. Considering carotenoids as an object of study, we demonstrate that the combined operation of the differential evolution algorithm and semiclassical quantum modeling of the optical response based on a generalized spectral density (the number of vibronic modes is arbitrary) allows us to distinguish quantum models of the pigment for different solvents. Moreover, it is determined that to predict the optical properties of monomeric pigments in protein, it is necessary to create a database containing, for each pigment, in addition to the absorption spectra measured in a predefined set of solvents, the parameters of the quantum model found using differential evolution.

## 1. Introduction

The development of methods for the non-invasive remote identification of organic pigments in living organisms is a complex and highly demanding task. Potentially, these methods, regardless of the specifics of their implementations, should combine at least two aspects: spectroscopy and computation. The spectroscopic aspect refers to an instrumentation framework with which high-resolution registration of the optical response is possible. The computational aspect involves the processing of the measurement results, modeling of the investigated spectra, and comparative analysis of the simulated and experimental results. The availability of an algorithm that would be able to distinguish the contribution of many components to the complex optical response of the investigated object would allow us to solve a large number of applied problems. In particular, the challenge of developing a methodology for detecting crops and plants infested with various parasitic organisms at early stages of their growth using spectroscopy in the visible and ultraviolet range is the focus of this study.

**Figure 1:**
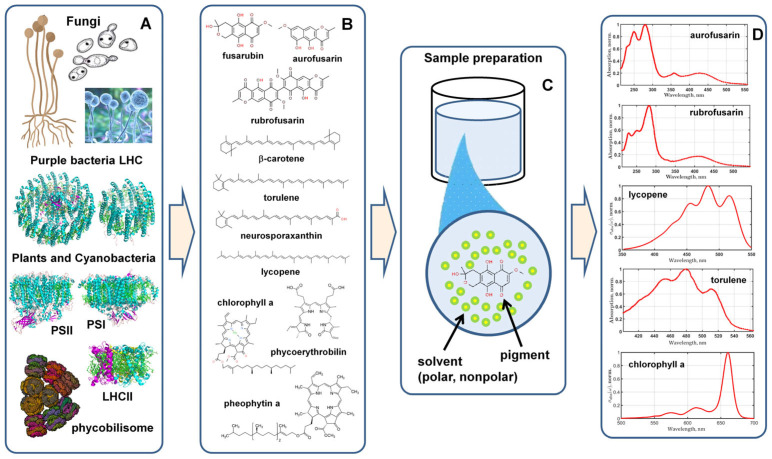
The protein complexes of fungi, bacteria, and plants contain pigments that are specific to each type of organism (**A**). To run the detection method, the database of spectra and the corresponding semiclassical quantum parameters have to be created for the pigments; (**B**) isolated from the fungi hyphae, the light harvesting complexes of bacteria and plants (PSI, PSII, LHCII, and, for example, phycobilisomes). All the spectra (**D**) must be measured for a predefined set of polar and nonpolar solvents (**C**).

Most organic pigments are part of the pigment–protein complexes of bacteria, plants, and fungal hyphae [1] (Figure 1A). Since pigments mainly absorb and fluoresce in the visible range, there is a potential opportunity to develop a technique to detect a pigment in any substance using its optical response. Spectroscopy methods currently provide a whole arsenal of tools for studying the optical properties of pigment molecules [2]: absorption and fluorescence spectroscopy [3] in the infrared, visible, and UV ranges [4], as well as Raman spectroscopy [5]. However, to perform measurements on the studied pigments (Figure 1B), they have to be isolated from proteins and placed in a cuvette with buffer (Figure 1A,C). In vivo measurements can be taken using the laser remote sensing technique [6,7,8,9,10], but the interpretation of the obtained signal is difficult [11] since the pigments are directly in the living organism and their optical properties are differ from those of pigments in solvents.

The main feature of the spectrum of any organic pigment is that its electronic absorption bands are quite broad (Figure 1D) and its line profile is usually irregularly shaped [12]. Such a spectrum cannot be fitted using simple Gaussians and Lorentzians, and therefore these easy methods of analysis do not work in this case. Complex absorption profiles, such as those of chlorophyll or carotenoids, are due to the presence of electron–phonon interaction between the vibrational modes of the molecular skeleton and the optically active electrons [13,14]. Moreover, all the spectra have inhomogeneity, which is the result of the effects of the immediate environment (protein, solvent, etc.) [15,16].

Using the example of the analysis of the optical properties of lycopene [17], one of the carotenoids found in fungi mycelia [18,19,20] and yeast cells [21], we demonstrate how the combined use of semiclassical quantum theory (the multimode Brownian oscillator model) and differential evolution (DE) [22,23], a multiparametric optimization algorithm [24], allows us to simultaneously fit the spectra of pigments in different solvents and obtain characteristic functions representing the effects of vibronic modes, proteins, and solvents on electronic excitation [25,26]. A key feature of our method is the use of generalized spectral density. In the calculation of the optical response within the multimode Brownian oscillator model, the spectral density completely determines the spectral width of the electronic transition and the phonon wing profile. For this purpose, it is necessary to specify a set of characteristic vibronic modes just before the spectrum calculation. However, if the evolutionary optimizer is used, then instead of a set of frequencies directly corresponding to the vibrational frequencies of the molecule, a generalized spectral density in the form of a comb of frequencies has to be provided to the input of the simulation procedure. While the method is running, unsuitable frequencies are filtered, and those that remain represent the actual fingerprint of the electronic transition of the pigment in a solvent or protein.

The article is structured as follows: in Section 2, the methodology of the data analysis and modeling of the pigment spectra is described in a formalized way; a brief overview of the optimization algorithm, the theoretical background of the simulation of absorption spectra, and some programming features and sample preparation are given in Section 3. The obtained results and prospects for further research are detailed in Section 4 and Section 5.

## 2. Statement of the Problem

**Figure 2:**
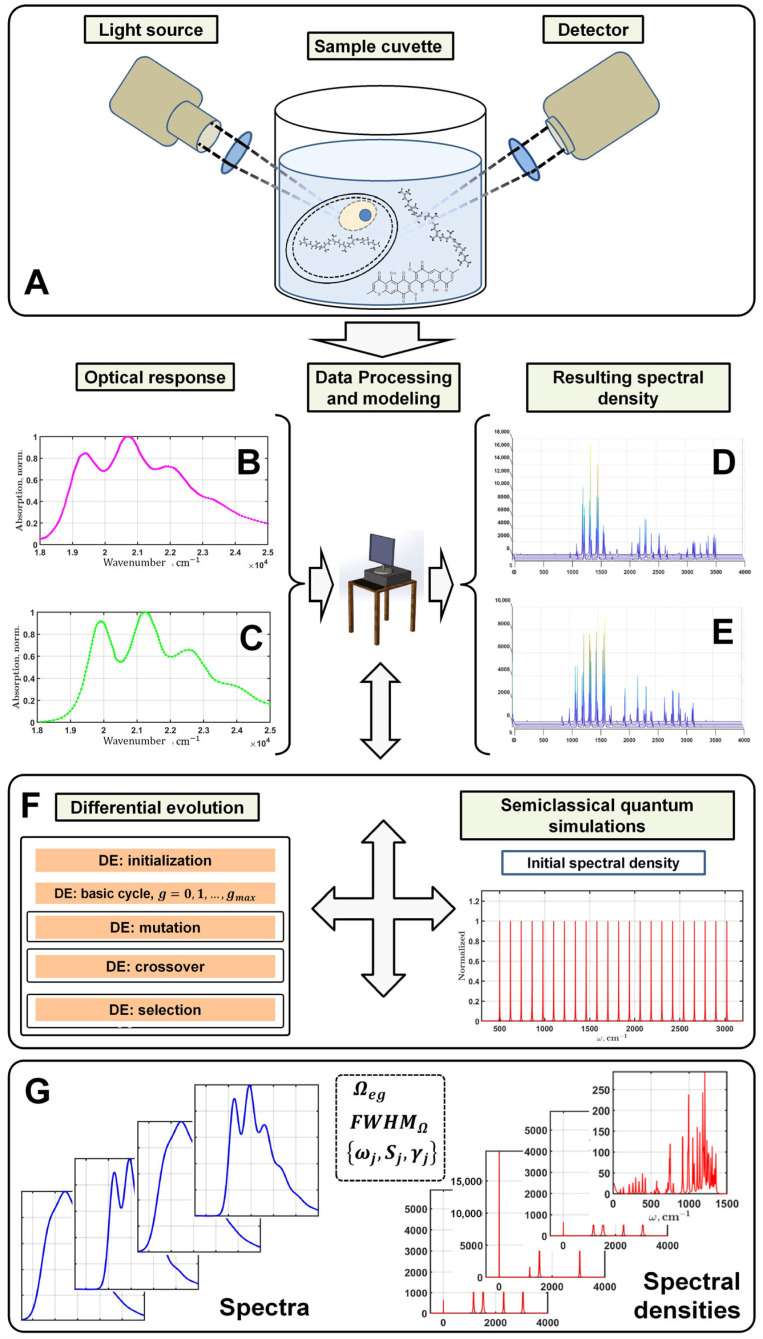
Graphic illustration explaining the main points of the methodology for the identification of pigments by their optical properties. Registration of the optical response includes a wide range of experimental techniques. (**A**) Absorption, Raman spectroscopy, remote sensing. Optical measurements (**B**,**C**), usually in the form of spectra; arrays of intensities and the corresponding frequencies at which they were measured are processed using multiparametric evolutionary optimization and procedures of spectra simulation within the framework of semiclassical quantum theory (**F**). The result of spectra processing is represented in the form of spectral densities (**D**,**E**), which are considered unique identifiers of pigments and their local environments as a system (coloring (**D**) and (**E**) represents the intensity of the peaks). These results are further compared and analyzed with accumulated data in a database (**G**) containing spectra and spectral densities for different pigments in reference solvents.

The basic idea of the method is visualized in Figure 2. The measured spectra are loaded into the software for processing and modeling (Figure 2B,C). The application of the multimode Brownian oscillator theory [27] for simulating the spectra of organic pigments in solvents or proteins from a mathematical point of view consists of a set of computational procedures, particularly the fast Fourier transform and numerical integration. These procedures process one-dimensional data arrays that are written in either frequency-domain or time-domain representation. Since only one electronic state is considered, the main optimization parameters are vibronic modes, on the basis of which correlation functions, and eventually the absorption spectrum profile, are calculated [25,26].

A special feature of our method is the initial representation of the spectral density (Figure 2F), which is an equidistant set of vibronic modes that interact equally with the electronic excited state. Thus, a set of model parameters (Figure 2G), including the electronic transition energy, the full width at half maximum of inhomogeneous broadening, and vibronic modes, is fed to the input of the simulation program. This set of parameters is called a model solution. Since the multimode Brownian oscillator theory is semiclassical and does not involve ab initio calculations [28,29], it is necessary to compare the calculated spectra with the experimentally measured ones in order to reach a proper modeling solution.

As a result, by varying the free parameters, one can try to find a solution for which the calculated spectra most accurately describe the measured ones. Ideally, the best solution is the set which gives an exact match between the calculated and measured spectra. Obviously, the process of finding the best model parameters can be optimized. The use of DE in this case is preferable to genetic algorithms because it allows us to vary the parameters continuously rather than discretely. Moreover, DE can classify the found solutions according to their cost values. In general, the algorithm may become stuck at a local minimum, but a special DE setting minimizes the probability of this event. Thus, the combined software implementation of the optical response modeling procedures and the differential evolution algorithm will allow us to find the exciton model parameters that provide the best agreement between the experimental and calculated data.

## 3. Materials and Methods

### 3.1. Differential Evolution

DE is a heuristic multiparametric evolutionary optimization method used to find the global minimum of a multimodal objective function. This method works effectively if it is necessary to minimize the functions with a large number of variables [22,23].

The classic algorithm works as follows: at the initialization stage, a population of candidate solutions (agents) is created inside n-dimensional space (where n is the number of parameters to be optimized). For each free parameter, a range of its possible values can be set. The best vector is selected from the others—the one for which the value of the objective function is the smallest. For the next generation, this vector becomes the base.

After that, operations of mutation, crossover, and selection occur to create the next generation of agents. Mutation is a linear operation for creating a new generation of vectors to which the best agent from the previous one can contribute. Depending on the agent’s contribution, there are two basic ways to create a mutant vector:

(1) $$v_{i}^{g}=x_{r0}^{g}+F\left(x_{r1}^{g}-x_{r2}^{g}\right),$$

(2) $$v_{i}^{g}=x_{best}^{g}+F\left(x_{r1}^{g}-x_{r2}^{g}\right),$$

where F∈[0,1] is the differential weight, which increases the diversity of the new generation of vectors.

When mutation is set, a crossover occurs, in which a trial vector is created by crossing the base and mutant vector. The crossover probability (Cr∈[0,1]) characterizes the number of parameters inherited from a mutant vector by the trial one. There are two types of crossover: exponential and binomial. Their names correspond to the type of distribution of the number of parameters inherited from the mutant vector. The choice of strategy, *F*, and *Cr* significantly affect the efficiency of the algorithm.

After crossover, the best trial vector of a new generation is compared with the best one from the previous generation. As in the principles of natural selection, the one with the smaller objective function will reach the next generation as the best vector. The number of generations can be set initially, or reaching a certain value of the objective function could be the stopping criterion.

The objective function characterizes the discrepancy between the experimental and simulated spectrum and is defined as follows:

(3) $$f\left(x_{i}^{g}\right)=\frac{1}{N}\sum_{n=1}^{N}{\left(I\left(\omega_{n}\right)-\sigma_{abs}\left(\omega_{n},x_{i}^{g}\right)\right)}^{2},$$

### 3.2. The Theory of Optical Response

**Figure 3:**
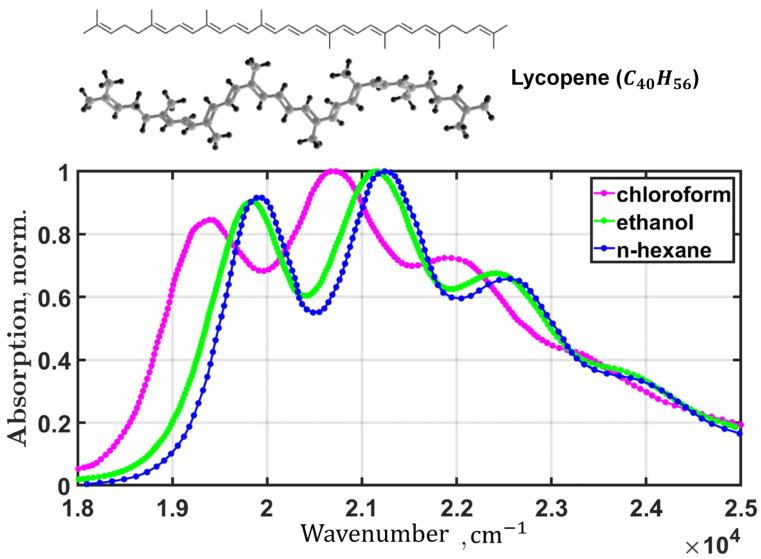
Chemical structure of the all-trans isomer of lycopene and absorption spectra in chloroform (magenta), ethanol (green), and n-hexane (blue) at room temperature.

Any effects caused by the interaction of matter with an electromagnetic field can be assessed by measuring a material quantity such as polarization. To demonstrate the functioning of our method, we used the absorption spectra of lycopene measured at room temperature in three solvents: chloroform, ethanol, and n-hexane (Figure 3).

Physical and chemical processes initiated by the absorption of a weak external field $E\left(r,t-t_{1}\right)$ by organic molecules are described by the first-order polarization component:

(4) $${P\left(r,t\right)}^{\left(1\right)}=-\frac{i}{\hbar }\int_{0}^{\infty }dt_{1}E\left(r,t-t_{1}\right)S^{\left(1\right)}\left(t_{1}\right),$$

Here, $S^{\left(1\right)}\left(t_{1}\right)$ is the first-order linear response function that includes the information of the material system. The general expression for the absorption spectrum of an electronic transition is

(5) $$\sigma_{abs}\left(\omega \right)=\int_{-\infty }^{\infty }dt\;S^{\left(1\right)}\left(t_{1}\right)e^{i\omega t}$$

To evaluate $S^{\left(1\right)}\left(t_{1}\right)$, the cumulant expansion method is applied [27]. This allows us to derive an exact solution for quantum systems using Gaussian statistics. By using this approach, $S^{\left(1\right)}\left(t_{1}\right)$ is calculated in terms of the correlation functions. By introducing the spectral density of the system under consideration, we obtain a simple numerical procedure for implementing a semiclassical theory for modeling the absorption spectra of organic pigments. The theoretical calculations are detailed in Appendix A.

### 3.3. Empirical Data

To make the whole method run, preliminary work is needed to create a database of semiclassical quantum parameters for as many pigments as possible involved in the metabolic process of parasitic organisms at different stages of their growth.

The sample preparation and measurements, which we consider optimal for the moment, are described in detail in [26]. Using astaxanthin as an example, whose spectra were measured in 18 polar and nonpolar solvents at room temperature from 350 nm to 600 nm, the corresponding sets of parameters to simulate the electronic transition of the pigment were obtained.

In addition to carotenoids, the database should include the processed spectra of other pigments typical for the fungi: fusarubin, aurofusarin, rubofusarin, β-carotene, torulene, neurosporaxanthin, and lycopene.

### 3.4. Programming and Software

At the current stage of developing a methodology for detecting infected crops and the accompanying software, we have used our previous designs for modeling the optical response. Procedures that can be used as independent library functions have been developed to estimate the spectral density, the lineshape function, and eventually the absorption spectrum profile. The implemented software package used to optimize the fitting of the spectra of organic pigments (chlorophylls, bacteriochlorophylls, and carotenoids) is described in the related publications. It includes a differential evolution procedure adapted for semiclassical quantum simulations (Figure 2F).

All the programs are written in C++, while the MKL library was used to speed up calculations with matrices and arrays.

## 4. Results

To demonstrate the functioning of our method, we used the absorption spectra of lycopene measured at room temperature in three solvents: chloroform, ethanol, and n-hexane (Figure 3). To simulate the spectra, the computational procedure sequentially evaluated Equations (A6)–(A8). The initial spectral density for each spectrum was calculated considering 30 vibronic modes. The frequencies, $\omega_{j}$, varied from 20 cm^−1^ to 3500 cm^−1^ in increments of 120 cm^−1^. The damping factors were up to 5 cm^−1^ for all modes [26]. Thus, the total number of parameters to be optimized, when the spectrum fit, was 35: the electronic transition energy, $\Omega_{eg}$; the full width at half maximum of inhomogeneous broadening, ${FWHM}_{\Omega }$; three parameters of the lowest vibronic mode, $\left\{\omega_{low},S_{low},\gamma_{low}\right\}$; and the Huang–Rhys factors for each vibronic mode of the spectral density function.

**Figure 4:**
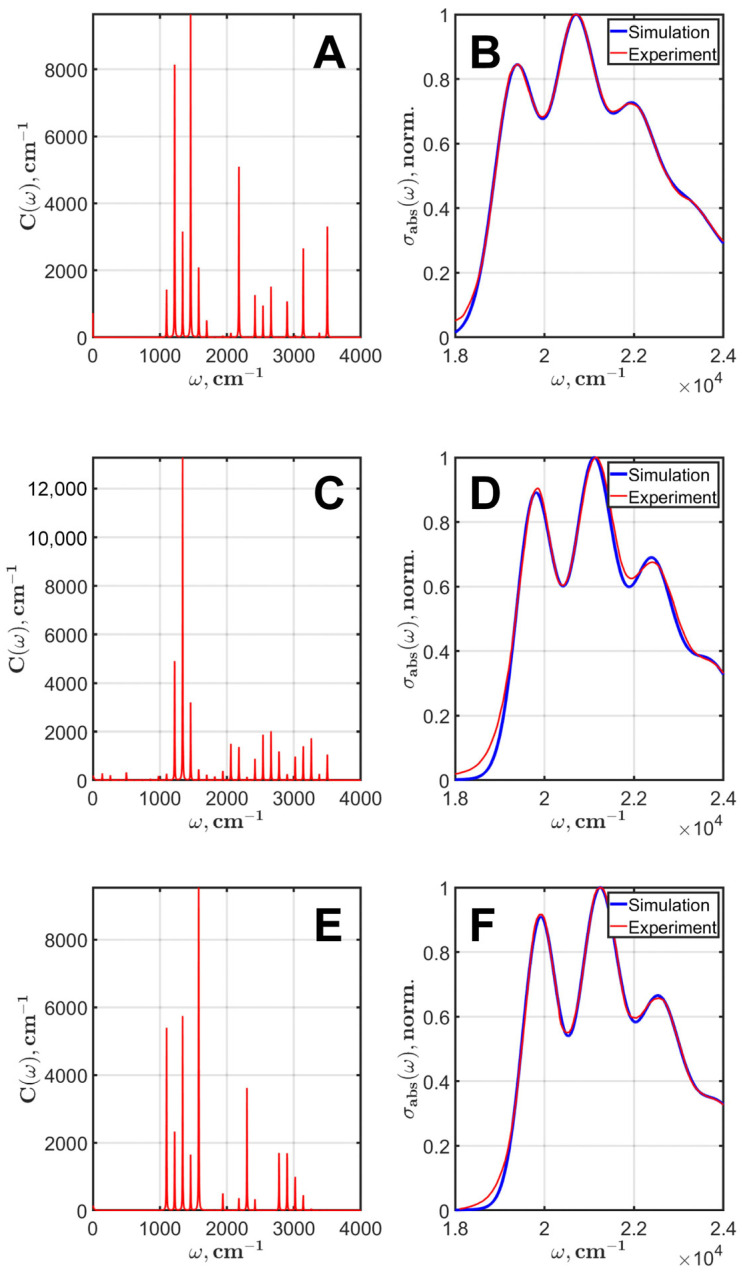
Results of the modeling of lycopene absorption spectra of the $\left|\left.S_{0}\right\rangle \to \right.\left|\left.S_{2}\right\rangle \right.$ electronic transition (**B**,**D**,**F**). The corresponding spectral densities in chloroform, ethanol, and n-hexane at room temperature (**A**,**C**,**E**).

DE optimization was carried out with the following settings: DE/best/1/bin strategy, *F* = 0.55, *Cr* = 0.9, the number of generations is 600. The results of modeling the lycopene absorption spectra (B,D,F) and spectral densities (A,C,E) in chloroform, ethanol, and n-hexane are shown in Figure 4.

To interpret the obtained spectral densities, it is convenient to divide them into three regions. The region from 800 cm^−1^ to 1500 cm^−1^ corresponds to the main vibronic modes of carotenoids; the region from 2000 cm^−1^ to 2500 cm^−1^ represents the double overtones of the $\nu_{2}$, $\nu_{3}$, and $\nu_{4}$ modes; and the region 3000 cm^−1^ and higher is the location of the double overtones of the $\nu_{1}$ mode.

Table 1 contains the full sets of parameters obtained after the best run of DE optimization. There is a clear tendency to zero out the Huang–Rhys factors of some modes that fall within a certain frequency domain. The results show the large influence of solvents on the spectra and the Huang–Rhys factor values.

We want to stress that the better the separation of the two overtone regions, the more accurate the results. For chloroform, the first overtone region strictly lies in the range from 2000 cm^−1^ to 2600 cm^−1^, and the second—from 3000 cm^−1^ to 3500 cm^−1^. There is a wide empty gap between them.

For n-hexane, a similar pattern is observed, but with a shift and a less wide gap (200 cm^−1^) between the overtone regions. The first overtone region corresponds to the range from 2000 cm^−1^ to 2400 cm^−1^, and the second region—from 2600 cm^−1^ to 3100 cm^−1^. Because of this, the agreement between the experimental and simulated spectra for n-hexane was slightly worse than for chloroform.

For ethanol, there is no clear gap between the two regions, resulting in the worst agreement between the experimental and simulated spectra of the three solvents. This may be due to both experimental errors and the special effect of this solvent on lycopene.

**Figure 5:**
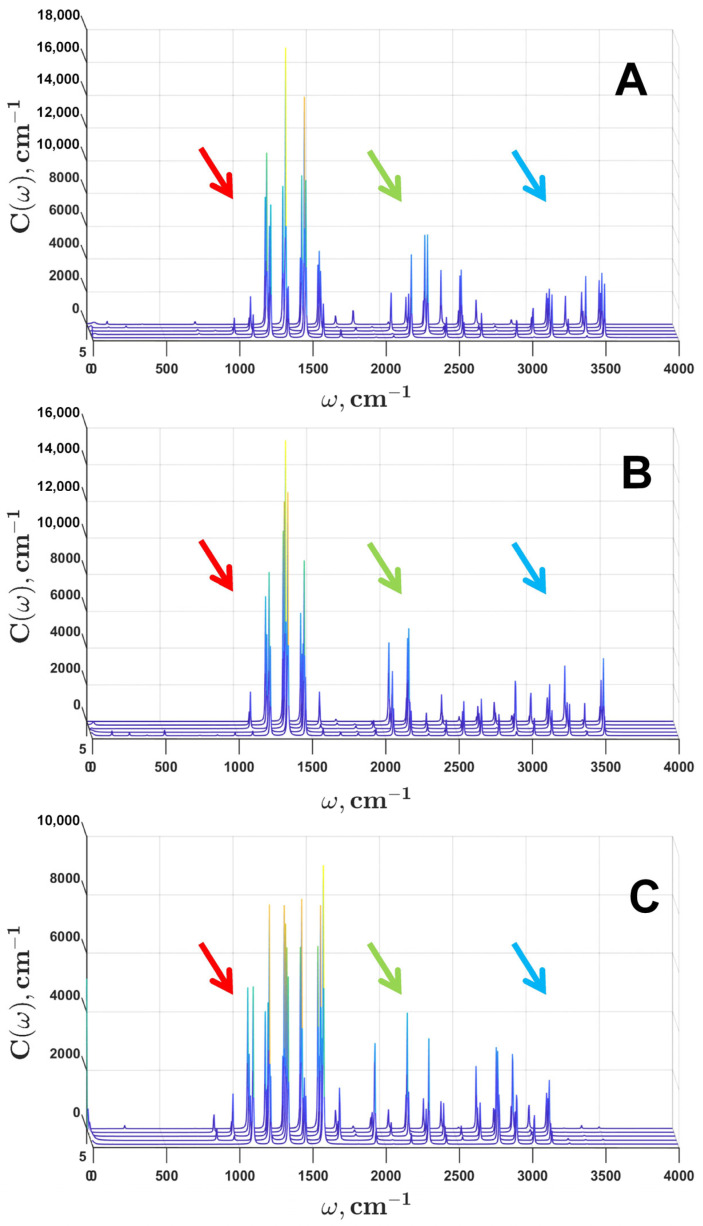
Spectral densities for lycopene in chloroform (**A**), ethanol (**B**), and n-hexane (**C**) obtained after five runs of optimization. Colored arrows indicate the range of $\nu_{1}$, $\nu_{2}$, $\nu_{3}$, and $\nu_{4}$ vibronic modes (red) of carotenoids and their overtones (green, blue).

The sets of spectral densities for lycopene in chloroform (A), ethanol (B), and n-hexane (C) obtained after five runs of DE optimization are shown on Figure 5. Red arrows point to the frequency region of the main carotenoid vibronic modes. Green and blue arrows point to the overtone regions.

As you can see, a set of statistics for each case creates a quite clear picture of the simulation results. For each solvent, the overtone regions have the same ranges with a small difference in mode intensity.

## 5. Discussion

### 5.1. Spectra Fitting

The idea of calculation using differential evolution is not new and has been successfully applied to astaxanthin [12,25,26], a keto-carotenoid produced by microalgae and the yeast fungus. Since we know all the information about the vibronic modes and their overtones, we were able to achieve results with high accuracy. However, in this work, we take a more general approach without limiting by a finite set of modes at certain frequencies. We set the comb of vibronic modes with a certain step over a wide range. On the one hand, such calculations become much longer due to the larger number of variables; on the other hand, with the correct implementation of the algorithm, the total accuracy can increase. For example, in the previously published study [30], there is a big discrepancy in the high-energy region because only two vibronic modes of carotenoids were used without overtones.

The idea of general spectral density is only feasible with the help of a powerful optimizer since it is not possible to solve the problem analytically. To increase the predictive power of the model, it is necessary to test it on a large number of carotenoids and other solvents, analyze the results obtained, and create a database. It is possible to find certain patterns based on the obtained results and classify the type of immediate surroundings according to their influence on the objects of study. It must be stressed that the accuracy of the results strongly depends on the step between the peaks in the comb (in this work, it was constant and equal to 120). This value needs fine-tuning optimization, because if the step is too large, many peaks may be missed, and if the step is too small, they may be indistinguishable from each other. Based on the available comb peaks, the final absorption spectrum can be calculated. However, the idea of general spectral density needs further detailed analysis.

Comparing our method with similar studies, it is first of all worth noting that most researchers apply data processing and machine learning (instead of evolutionary optimization) to plant stress detection and automatic discrimination of crops and weeds [31,32,33]. The advantage of our method is that we do not require knowledge about the leaf morphology (shape, color, size) [31,32]; our goal is to detect the presence of characteristic pigments by their optical response. Since some pigments are specific to the particular stage of fungi growth, our methodology allows us to recognize the disease at an early stage, when the external signs of infection are not yet visible. In addition, it is obvious that from the analysis of photographs alone, it is impossible to obtain a description of the physical and chemical processes occurring directly in the leaves or seeds. Also, input data containing a large number of images are more time-consuming from the point of view of computer processing than mathematical data (spectra, tables).

### 5.2. Possiblity of Application for the Early Detection of Fungi in Crops and Agricultural Products

There are a large number of fungal diseases affecting various cereal crops such as oats, wheats, barley, and maize [34,35]. Moreover, infection can occur at any stage of crop development [36] and does not depend on the volume and quality of the harvest [37]. These contagious diseases are caused by fungi of the genera *Fusarium, Alternaria, Neuraspora*, and many others [38,39]. It should be noted that most fungi produce and accumulate mycotoxins that are dangerous for humans and animals [40]. Therefore, the study of the characteristic symptoms of the disease and the improvement of diagnostic methods is a crucial practical task [41,42]. One of the distinctive signs indicating the presence of a certain species of fungus on the outer face of seeds is the appearance of color spots [43,44,45] due to the fact that fungi have pigment molecules. The number of pigments synthesized by fungi is very large [46]; the mycelium of some species at different states of their growth may contain up to a dozen pigments. Thus, the idea of detecting the plant pathogens using optical methods [47] looks promising. Naturally, quantum chemistry calculations applying the TD-DFT approach have been used for many carotenoids and other photosynthetic pigments [48,49]; moreover, some studies have recently been published on the simulation of the vibrational spectrum of rubrofusarin [50]. It is worth noting that these methods can estimate the vibrational structures of the ground state quite well; however, for modeling excited states, complicating the calculations is necessary.

The developed method for detecting pigments in the immediate surroundings might also be used to recognize early crop infestation, both via remote sensing and when the samples for study are washed off the surface of the grains. It is for such practical applications that the use of a database containing pigment spectra and generalized spectral density functions plays a key role. The generalized spectral density calculated for pigment spectra in different solvents allows us to distinguish frequencies, the influence of which does not actually change from the pigment environment, as well as those that are specific to a given environment. Such classification of spectral density frequencies will enable us to examine the spectral density obtained for spectra not included in the database and to draw conclusions about the belonging of the investigated spectra to a particular pigment. The main question is whether it is possible to obtain the parameters with sufficient accuracy using spectral density as the characteristic of the electronic transition of the observed spectrum. We will try to clarify this problem in our future studies on this topic.

## 6. Conclusions

In this work, we have presented a prototype method that allows us to analyze the optical response of organic pigments both in solvents and in a protein environment. Potential applications include the identification of different microorganisms, based on the signal of the pigments that are part of their cell membranes. In particular, it could be possible to detect the infection of agricultural crops by parasitic fungi at early stages of their growth.

The technique is based on the combined usage of semiclassical quantum theory for modeling the optical response and a multiparametric evolutionary optimization algorithm. The synergistic effect of such a combination is manifested in the possibility to predict the optical properties of organic pigments depending on the immediate protein environment, based on the results of modeling these pigments in reference media (usually polar and nonpolar solvents).

With the example of lycopene in three solvents (chloroform, ethanol, and n-hexane), we showed that the use of spectral density of a special kind (in the form of a comb with an equidistant distribution of vibronic modes from 20 cm^−1^ to 3500 cm^−1^) as an initial condition for multiparametric optimization allowed us to fit the experimental data with great accuracy and, at the same time, obtain statistically distinguishable values of the resulting spectral density. Thus, for the spectra of lycopene in different solvents, characteristic frequencies in the spectral density were identified, which unambiguously determined the solvent in which the spectrum was measured.

Obviously, the creation of a database of optical spectra measured in reference solvents for widely distributed pigments will allow the generation of characteristic spectral densities, on the basis of which it will be possible to recognize signals from pigments in living organisms in the future.

## Figures and Tables

**Table 1:** Parameters of the multimode Brownian oscillator model used to simulate absorption spectra of lycopene in chloroform, ethanol, and n-hexane at room temperature.

	Lycopene		
	Chloroform	Ethanol	n-Hexane
$\Omega_{eg},\;{\mathrm{cm}}^{-1}$	21,526.7	21,833.2	21,899.4
${FWHM}_{\Omega },\;{\mathrm{cm}}^{-1}$	1090.0	1042.3	845.2
$\omega_{low},\;{\mathrm{cm}}^{-1}$	23.5	12.0	36.9
$S_{low}$	1.387	0.215	1.745
$\gamma_{low},\;{\mathrm{cm}}^{-1}$	490.1	314.4	115.7
$S_{20}$	0.026	0.075	0.125
$S_{140}$	$0.000\;(<10^{-3}$)	$0.000\;(<10^{-3}$)	0.001
$S_{260}$	$0.000\;(<10^{-3}$)	$0.000\;(<10^{-3}$)	$0.000\;(<10^{-3}$)
$S_{380}$	0.001	$0.000\;(<10^{-3}$)	$0.000\;(<10^{-3}$)
$S_{500}$	$0.000\;(<10^{-3}$)	$0.000\;(<10^{-3}$)	$0.000\;(<10^{-3}$)
$S_{620}$	$0.000\;(<10^{-3}$)	$0.000\;(<10^{-3}$)	$0.000\;(<10^{-3}$)
$S_{740}$	$0.000\;(<10^{-3}$)	$0.000\;(<10^{-3}$)	$0.000\;(<10^{-3}$)
$S_{860}$	$0.000\;(<10^{-3}$)	$0.000\;(<10^{-3}$)	$0.000\;(<10^{-3}$)
$S_{980}$	$0.000\;(<10^{-3}$)	$0.000\;(<10^{-3}$)	$0.000\;(<10^{-3}$)
$S_{1100}$	0.103	$0.000\;(<10^{-3}$)	0.389
$S_{1220}$	0.457	0.311	0.131
$S_{1340}$	0.146	0.673	0.267
$S_{1460}$	0.393	0.101	0.067
$S_{1580}$	0.078	$0.000\;(<10^{-3}$)	0.359
$S_{1700}$	0.019	0.002	$0.000\;(<10^{-3}$)
$S_{1820}$	0.001	$0.000\;(<10^{-3}$)	$0.000\;(<10^{-3}$)
$S_{1940}$	0.001	0.050	0.015
$S_{2060}$	0.003	0.084	$0.000\;(<10^{-3}$)
$S_{2180}$	0.096	0.017	0.001
$S_{2300}$	$0.000\;(<10^{-3}$)	$0.000\;(<10^{-3}$)	0.058
$S_{2420}$	0.018	0.004	0.005
$S_{2540}$	0.013	$0.000\;(<10^{-3}$)	$0.000\;(<10^{-3}$)
$S_{2660}$	0.020	0.026	$0.000\;(<10^{-3}$)
$S_{2780}$	$0.000\;(<10^{-3}$)	0.014	0.023
$S_{2900}$	0.015	$0.000\;(<10^{-3}$)	0.023
$S_{3020}$	0.001	0.005	0.012
$S_{3140}$	0.027	0.009	0.005
$S_{3260}$	$0.000\;(<10^{-3}$)	0.028	$0.000\;(<10^{-3}$)
$S_{3380}$	0.001	0.003	$0.000\;(<10^{-3}$)
$S_{3500}$	0.023	$0.000\;(<10^{-3}$)	$0.000\;(<10^{-3}$)

## Data Availability

Data are contained within the article.

## Appendix A

Let us consider a two-level system consisting of ground $\left|\left.g\right\rangle \right.$ and excited $\left|\left.e\right\rangle \right.$ electronic states. The excited electronic states of carotenoids in the 400–600 nm region corresponds to the so-called optically allowed $\left|\left.S_{0}\right\rangle \to \right.\left|\left.S_{2}\right\rangle \right.$ transition. The complete Hamiltonian of the system, including the sets of vibrational modes, is written as:

(A1) $$H_{tot}={\left|\left.g\right\rangle \right.H}_{g}\left(q\right)\left.\left\langle g\right.\right|+\left|\left.e\right\rangle \right.H_{e}\left(q\right)\left.\left\langle e\right.\right|,$$

(A2) $$H_{g}\left(q\right)=\sum_{j}^{N}\left(\frac{p_{j}^{2}}{2m_{j}}+\frac{1}{2}m_{j}\omega_{j}^{2}q_{j}^{2}\right),$$

(A3) $$H_{e}\left(q\right)=\hbar \omega_{eg}^{0}+\sum_{j}^{N}\left(\frac{p_{j}^{2}}{2m_{j}}+\frac{1}{2}m_{j}\omega_{j}^{2}{\left(q_{j}+d_{j}\right)}^{2}\right),$$

where $H_{g}\left(q\right)$ and $H_{e}\left(q\right)$ are the Hamiltonians of the ground and excited states; $\omega_{eg}^{0}$ is the energy gap between $\left|\left.g\right\rangle \right.$ and $\left|\left.e\right\rangle \right.$; $p_{j}$, $m_{j}$, $\omega_{j}$, and $q_{j}$ are the effective moments, masses, frequencies, and coordinates of the vibronic modes; $d_{j}$ is the displacement parameter characterizing the deformation of the $\left|\left.e\right\rangle \right.$ potential curve; and *N* is the number of modes.

The theory assumes that each vibronic mode of the system described by $H_{tot}$ interacts with a set of bath modes. This assumption allows us to take into account the contribution of low and high frequencies of nuclear oscillations in one equation for the spectral density function. Thus, $H_{tot}$ is modified by adding the $H_{VB}$ contribution:

(A4) $$H_{tot}=H_{g}+H_{e}+H_{VB},$$

(A5) $$H_{VB}=\sum_{n}^{M}\left[\frac{p_{n}^{2}}{2m_{n}}+\frac{1}{2}m_{n}\omega_{n}^{2}x_{n}^{2}-x_{n}\sum_{j}c_{nj}q_{j}+\frac{\sum_{j}c_{nj}^{2}q_{j}^{2}}{2m_{n}\omega_{n}^{2}}\right],$$

Here, *M* enumerates the bath modes; $p_{n}$, $m_{n}$, and $\omega_{n}$ are the parameters of the *n*th bath mode; and $c_{nj}$ is the effective interaction between the nth bath mode and the *j*th vibronic mode.

Since we are interested in the linear optical response, the system described by the Hamiltonians (A4) and (A5) must be in the ground state $\rho_{g}=\exp\left(-\beta H_{g}\right)/Tr\exp\left(-\beta H_{g}\right)$, and 1/β=kT, where *T* is the temperature. Here, $\rho \left(-\infty \right)=\left|\left.g\right\rangle \rho_{g}\right.\left.\left\langle g\right.\right|$ is the electronic density operator $\rho \left(t\right)$ in the thermal equilibrium conditions.

There are several ways to evaluate the spectral density function $C^{\prime \prime }\left(\omega \right)$; one of them is the path integral approach. An expression, suitable for numerical calculations, is as follows:

(A6) $$C^{\prime \prime }\left(\omega \right)=\sum_{j}\frac{2S_{j}\omega_{j}^{3}\omega \gamma_{j}}{{\left(\omega_{j}^{2}-\omega^{2}\right)}^{2}+\omega^{2}\gamma_{j}^{2}},$$

It already contains a set of effective parameters $\left\{\omega_{j},S_{j},\gamma_{j}\right\}$ that is used to simulate the optical response instead of the parameters from (A2), (A3), and (A5). The electron–phonon coupling energy between an electronic state and the jth mode is described by $S_{j}$, the Huang–Rhys factor, which is proportional to $d_{j}$ in (A3); $\gamma_{j}$ is the damping factor of the jth vibronic mode. The expression for the spectral density is used to calculate the $g\left(t\right)$ lineshape function:

(A7) $$g\left(t\right)=\frac{1}{2\pi }\int_{-\infty }^{\infty }d\omega \frac{1-\cos\omega t}{\omega^{2}}\coth\left(\beta \hbar \omega /2\right)C^{\prime \prime }\left(\omega \right)-\frac{i}{2\pi }\int_{-\infty }^{\infty }d\omega \frac{\sin\left(\omega t\right)-\omega t}{\omega^{2}}C^{\prime \prime }\left(\omega \right),$$

$g\left(t\right)$ is the temperature-dependent function that carries information about the contribution of each vibronic mode to the final absorption spectrum. The broadening of the spectrum associated with the vibronic modes of a pigment is called a homogeneous one. To make the simulated spectrum appear as realistic as possible, in addition to inhomogeneous broadening, it is necessary to take into account the influence of the immediate surroundings of the molecule. Thus, the final expression for the absorption profile is:

(A8) $$\sigma_{abs}\left(\omega \right)=\frac{1}{\pi }Re\;\int_{0}^{\infty }dte^{i\left(\omega -\Omega_{eg}\right)t}e^{-g\left(t\right)}e^{-\frac{1}{2}{\left(\Delta t\right)}^{2}},$$

where $\Omega_{eg}$ is the energy of the $\left|\left.g\right\rangle \right.\to \left|\left.e\right\rangle \right.$ electronic transition, $\Delta =FWHM/2\sqrt{2\cdot ln2}$ is the standard deviation of the inhomogeneous broadening, and ${FWHM}_{\Omega }$ is the full width at half maximum.
